# Fermentation of Mannitol Extracts From Brown Macro Algae by Thermophilic *Clostridia*

**DOI:** 10.3389/fmicb.2018.01931

**Published:** 2018-08-20

**Authors:** Theo Chades, Sean M. Scully, Eva M. Ingvadottir, Johann Orlygsson

**Affiliations:** Faculty of Natural Resource Sciences, University of Akureyri, Akureyri, Iceland

**Keywords:** third generation biomass, bioethanol, *Thermoanaerobacter*, seaweed, bioprocessing, extremophiles, *Ascophyllum nodosum*, *Laminaria digitata*

## Abstract

Mannitol-containing macro algae biomass, such as *Ascophyllum nodosum* and *Laminaria digitata*, are a potential feedstock for the production of biofuels such as bioethanol. The purpose of this work was to evaluate the ability of thermophilic anaerobes within Class *Clostridia* to ferment mannitol and mannitol-containing algal extracts. Screening of the type strains of six genera, *Caldanaerobius, Caldanaerobacter, Caldicellulosiruptor, Thermoanaerobacter, Thermobrachium*, and *Thermoanaerobacterium*) was conducted on 20 mM mannitol and revealed that 11 of 41 strains could utilize mannitol with ethanol being the dominant end-product. Mannitol utilization seems to be most common within the genus of *Thermoanaerobacter* (7 of 16 strains) with yields up to 88% of the theoretical yield in the case of *Thermoanaerobacter pseudoethanolicus*. Six selected mannitol-degrading strains (all *Thermoanaerobacter* species) were grown on mannitol extracts prepared from *A. nodosum* and *L. digitata*. Five of the strains produced similar amounts of ethanol as compared with ethanol yields from mannitol only. Finally, *T. pseudoethanolicus* was kinetically monitored using mannitol and mannitol extracts made from two macro algae species, *A. nodosum* and *L. digitata* for end-product formation.

## Introduction

Due to the worldwide energy crisis and environmental problems associated with the utilization of petroleum products, the need for biofuel production from renewable feedstocks that do not compete with agriculture has been of increased interest over the past several decades (Sánchez and Cardona, 2008; Scully and Orlygsson, 2015). While the production of bioethanol from lignocellulosic biomass has made advances in terms of pretreatment of the biomass and the development of microorganisms well-suited to this task, substantially less work has been done on the utilization of marine biomass, such as macro algae, as a raw material. The utilization of macro algae (third generation biomass) as a feedstock is of special interest as marine biomass often has high productivity and does not compete with arable land (Daroch et al., 2013; Wei et al., 2013). The utilization of third generation biomass remains challenging due to the diversity of chemical compositions of macro algae and as well as lack of well-established methodologies for the saccharification of this type of biomass (Daroch et al., 2013). While macro algae do not contain lignin like terrestrial plants, many macro algae contain phlorotannins which are similarly structured to lignin in that it is composed of phloroglucinol, an aromatic molecule (Kawai and Murata, 2016). Brown macro algae have complex carbohydrate composition containing alginate, fucoidan, mannitol, and laminarin (Wei et al., 2013; Xia et al., 2015; Kawai and Murata, 2016). Mannitol and laminarin function as reserve carbohydrates and are accumulated during summer months of the northern hemisphere (Adams et al., 2011) reaching concentrations as high as 25–30% on a dry weight basis (Daroch et al., 2013; Wei et al., 2013). Brown macroalgae are harvested commercially in large quantities in Asia and Europe (Muty and Banerjee, 2012). In 2012, 24 million tons of macro algae were harvested globally with only a handful of nations responsible indicating that there is great potential to exploit these materials (Radulovich et al., 2015). In the west of Iceland, *Laminaria digitata* and *Ascophyllum nodosum* have been dried using geothermal heat since the 1970s which was commercialized by Thorverk, ehf in the mid-1980s although harvested quantities are less than 20 tons per year. As a raw material for bioprocessing, brown macro algae have been used for biogas production while for bioethanol production the complexity of the biomass still presents a serious challenge for economic success due to the diversity of carbohydrates present as well as pretreatment methodologies still being in their infancy (Wei et al., 2013; Enquist-Newman et al., 2014; Milledge et al., 2014; Jiang et al., 2016).

Thermophilic species within Class *Clostridia*, including genera such as *Caldicellulosiruptor, Thermoanaerobacter*, and *Thermoanaerobacterium*, have been isolated from a wide range of thermal environments including many in terrestrial geothermal areas in Eurasia including Iceland, and the Kamchatka Peninsula, Russia (Burgess et al., 2007). As with thermoanaerobes in general, work at higher operating temperatures improves substrate solubility and reduces viscosity, improves the thermodynamics of many fermentative processes, and could potentially allow for *in situ* distillation of volatile end products such as ethanol. Many thermophilic *Clostridia* are of interest for their inherent tolerance to extreme environmental conditions and their ability to produce potential biofuels from a broad range of substrates including monosaccharides present in lignocellulosic biomass although highly reduced sugar alcohols have received little attention. Many of these thermophilic *Clostridia* also have the ability to degrade polymeric carbohydrates which often includes starch, xylan, and even crystalline cellulose (Ren et al., 2009; Taylor et al., 2009; Chang and Yao, 2011; Scully and Orlygsson, 2015). Several *Caldicellulosiruptor* species in particular are highly versatile bioprocessing platforms with diverse capabilities involving the deconstruction of complex carbohydrates including cellulose and hemicelluloses (Blumer-Schuette et al., 2012; Carere et al., 2012). Due to the broad substrate spectra and diverse metabolic capabilities demonstrated by the aforementioned thermophilic anaerobes, they make natural prospecting candidates for the utilization of algal biomass, particularly mannitol. While the utilization of sugar alcohols, such as D-mannitol, are often reported in the work describing novel species, end product spectra are seldom reported.

Sugar alcohols, such as mannitol, are naturally occuring or can be produced by the catalytic dehydrogenation of their correpsonding hexose or pentose or can be produced by yeast during fermentation (Tanghe et al., 2005). Mannitol, in addition to being an inexpensive substrate, can be easily isolated from brown algae such as *A. nodosum, Laminaria* species, and *Macrocystis pyrifera*, such as sorbitol (glucitol), and mannitol. Beyond their applications as artificial sweeteners and laxatives, other potential applications of mannitol include clinical applications and as a fermentation feedstock (Daroch et al., 2013; Wei et al., 2013). Mannitol is interesting for biofuel production since its heating value is higher than that of glucose (3025 kJ/mol versus 2805 kJ/mol) and is a more reduced fermentation substrate as compared to hexoses and thus is an unexploited resource for bioethanol production (Daroch et al., 2013; Wei et al., 2013).

Mannitol utalization is a fairly ubiquitous trait with organisms including yeast such as *Candida albicans* and *Pichia angophorae* (Horn et al., 2000) although some strains of *Saccharomyces cerevisiae* have been adapted to utilize mannitol (Quain and Boulton, 1987). Mesophilic bacteria capable of mannitol utilization include *Staphylococcus aureus, Escherichia coli* (Kim et al., 2011), *Zymobacter palmae* (Horn et al., 2000), *Vibrio tritonius* (Matsumura et al., 2014), *Clostridium butyricum* (Heyndrickx et al., 1989), *Clostridium difficile* (Kazamias and Sperry, 1995), *Clostridium acetobutylicum* (Kaid et al., 2016), and *Anaerobium acetethylicum* (Patil et al., 2015). Thermophilic bacteria known to degrade mannitol anaerobically include the recently described *Defluviitalea phaphyphila* which is capable of degrading multiple macro algae components (Ji et al., 2016a,b), *Caldicoprobacter algeriensis* (Bouanane-Darenfed et al., 2011), *Thermoanaerobacter wiegelii* which yields ethanol (Cook et al., 1996), and *Thermoanaerobacter pentosaceus* (Sittijunda et al., 2013; Tomás et al., 2013).

As brown algae commonly contain high concentrations of mannitol, commercially harvested brown algae species such as *L. digitata* and *A. nodosum* make logical choices of raw material. *Macrocystis pyrifera* contains up to 12.4% mannitol by weight (Mateus et al., 1977) while the mannitol content in *L. digitata* has been found to vary between 3 and 21% (Black et al., 1952) although more recent reports have reported results above 30% on a weight basis (Adams et al., 2011). The extraction of mannitol from brown algae can be accomplished using solid-liquid extraction using weakly acidic solutions as has been reported for brown algae such as *Macrocystis pyrifera* (Mateus et al., 1977).

The present work examines the extraction of mannitol from macro algae and its subsequent fermentation to ethanol by thermophilic *Clostridia*. Comparisons between dilute acid solid-liquid extraction conditions under mild (50°C or less) conditions were performed. The ability of thermophilic *Clostridia* in the genera of *Caldanaerobius, Caldicellulosiruptor, Caldanaerobacter, Thermoanaerobacterium, Thermoanaerobacter*, and *Thermobrachium* to utilize mannitol as the sole substrate as well as mannitol extracted by dilute acid extraction from two ubiquitous mannitol-containing brown algae species, *A. nodosum* and *L. digitata* were tested. A kinetic experiment with a promising strain, *Thermoanaerobacter pseudoethanolicus*, was also performed. This study demonstrates the ease with which mannitol can be extracted and fermented to bioethanol from readily available brown algae.

## Materials and Methods

### Strains and Cultivation Conditions

Organisms were cultivated in Basal Mineral (BM) medium prepared as previously described (Sveinsdottir et al., 2009) with modifications; the medium consisted of (per liter): NaH_2_PO_4_⋅2H_2_O 3.04 g, Na_2_HPO_4_⋅2H_2_O 5.43 g, NH_4_Cl 0.3 g, NaCl 0.3 g, CaCl_2_⋅2H_2_O 0.11 g, MgCl_2_ × 6H_2_O 0.1 g, yeast extract 2.0 g, resazurin 1 mg, trace element solution 1 mL, vitamin solution (DSM141) 1 mL, and NaHCO_3_ 0.8 g. The trace element solution consisted of the following on a per liter basis: FeCl_2_ × 4H_2_O 2.0 g, EDTA 0.5 g, CuCl_2_ 0.03 g, H_3_BO_3_, ZnCl_2_, MnCl_2_ × 4H_2_O, (NH_4_)Mo_7_O_24_, AlCl_3_, CoCl_2_ × 6H_2_O, NiCl_2_, and 0.05 g, Na_2_S × 9H_2_O 0.3 g, and 1 mL of concentrated HCl. The final mannitol concentration was 20 mM in all cases. The medium was prepared by adding the buffer to distilled water containing resazurin and boiled for 10 min and cooled under nitrogen flushing (<5 ppm O_2_). The mixture was then transferred to serum bottles using the Hungate technique (Hungate, 1969; Miller and Wolin, 1974) and autoclaved for 60 min. Media preparations containing algal extracts were sterilized by Tindallization to avoid browning of the media; these serum bottles were heated twice at 90°C for 60 min. All other components of the medium were added separately through filter (0.45 μm) sterilized solutions. All experiments were conducted at 65°C and at pH of 7.0 with a liquid-gas phase (L-G) ratio of 1:1 in which the gas phase consists of nitrogen. In all cases, experiments were performed in triplicate.

Forty one strains of thermophilic anaerobes from the genera of *Caldicellulosiruptor, Caldanaerobacter, Thermoanaerobacter*, and *Thermoanaerobacterium* were purchased from Deutsche Sammlung von Mikroorganismen und Zellkulturen (DSMZ); *Thermoanaerobacterium thermostercoris* (DSM 22141), *Thermoanaerobacterium thermosulfurigenes* (DSM 2229), *Thermoanaerobacterium aotearoense* (DSM 10170), *Thermoanaerobacterium thermosaccharolyticum* (DSM 571), *Thermoanaerobacterium saccharolyticum* (DSM 7060), *Thermoanaerobacterium xylanolyticum* (DSM 7097), *Thermoanaerobacterium aciditolerans* (DSM 16487), *Caldanaerobius polysaccharolyticus* (DSM 13641), *Caldanaerobius zeae* (DSM 13642), *Caldanaerobius fijiensis* (DSM 17918), *Thermobrachium celere* (DSM 13655), *Caldicellulosiruptor changbaiensis* (DSM 26941), *Caldicellulosiruptor saccharolyticus* (DSM 8903), *Caldicellulosiruptor owensis* (DSM 13100), *Caldicellulosiruptor bescii* (DSM 6725), *Caldicellulosiruptor acetigenus* (DSM 7040), *Caldicellulosiruptor lactoaceticus* (DSM 9545), *Caldicellulosiruptor kristjanssonii* (DSM 12137), *Caldicellulosiruptor hydrothermalis* (DSM 18901), *Caldicellulosiruptor kronotskiensis* (DSM 18902), *Thermoanaerobacter acetoethylicus* (DSM 2359), *Thermoanaerobacter brockii* subsp. *brockii* (DSM 1457), *T. brockii* subsp. *finnii* (DSM 3389), *T. brockii* subsp. *lactiethylicus* (DSM 9801), *Thermoanaerobacter italicus* (DSM 9252), *Thermoanaerobacter ethanolicus* (DSM 2246), *Thermoanaerobacter kivui* (DSM 2030), *Thermoanaerobacter mathrani* subsp. *mathrani* (DSM 11426), *T. pentosaceus* (DSM 25963), *T. pseudoethanolicus* (DSM 2355), *Thermoanaerobacter siderophilus* (DSM 12299), *Thermoanaerobacter sulfurigignens* (DSM 17917), *Thermoanaerobacter sulfurophilus* (DSM 11584), *Thermoanaerobacter thermohydrosulfuricus* (DSM 567), *Thermoanaerobacter uzonensis* (DSM 18761), *T. wiegelii* (DSM 10319), *Caldanaerobacter subterraneus* subsp. *yonseiensis* (DSM 13777), *C. subterraneus* subsp. *subterraneus* (DSM 13054), *C. subterraneus* subsp. *pacificus* (DSM 12653), *C. subterraneus* subsp. *tengcongensis* (DSM 15242), and *Caldanaerobacter uzonensis* (DSM 18923). All cultivations were conducted at pH 7.0 at the organism’s T_opt_ which is listed in **Supplementary Table S1**.

All inoculation stocks of the strains were taken from frozen (-20°C) cultures with rigorously degassed 30% (v/v) glycerol and reactivated on BM medium containing glucose (20 mM). Reactivated cultures were inoculated (2% v/v) from exponential growth phase to 25 mL serum bottles (liquid-gas ratio 1:1). Cultures were grown for 7 days and screened for end-product formation.

### Macro Algae Collection and Preparation

Multi-kilogram quantities of brown algae were collected in the summer of 2015. *A. nodosum* was obtained from the shores just north of Akureyri, Iceland (65°41′53.31′′N, 18°6′32.80′′W) in July of 2015. *L. digitata* was obtained from a coastal area north of Husavik, Iceland (66°3′38.64′′N, 17°21′18.92′′W) in August of 2015. Collected samples were transported to the laboratory and briefly washed with cold tap water; material was then dried in an oven at 45°C for 48 h. Dried algae was milled in a Waring blender and then milled to less than 2 μm in a Herzog HSM 50 pulverizing mill. The resultant algal meal was stored in air tight containers prior to use.

### Extraction of Mannitol From Macro Algae

The solid-liquid extraction of mannitol from *A. nodosum* and *L. digitata* was investigated by varying both temperature (0, 25, 50°C) and HCl concentration (0, 50, 100 mM). One gram of algal meal was placed in a 50 mL polypropylene tube with 10 mL of extraction solution (0, 0.05 M, or 0.1 M HCl) and mixed at 250 rpm for 15 min at a 45° angle to ensure thorough mixing. The supernatant was collected by centrifugation (4700 rpm, 15 min, 4°C). This was repeated for a total of four extraction. Extraction solutions were stored at -40°C prior to analysis for mannitol, total phenolics, and protein.

### Extraction Kinetics of Algal Meal

10 g of algal meal was extracted in triplicate on a 10 g scale with 100 mL of extraction solution (50 mM HCl for *A. nodosum* or dH_2_O for *L. digitata*) based on results of the previous experiment. Samples were mixed at 250 rpm and supernatant was withdrawn over 15 min in 3 min intervals, centrifuged at 13,000 rpm, and stored at -40°C prior to analysis for mannitol, total phenolics, and protein.

### Large-Scale Extraction of Mannitol From Algal Meal

100 g of algal meal was extracted with 1 L of either 50 mM HCl (*A. nodosum*) or dH_2_O (*L. digitata*) at 200 rpm for 10 min at 25°C. The extract was centrifuged at 4700 rpm for 15 min at 4°C. 10 g of CaCl_2_ was then added to precipitate alginate present and the resultant solution was centrifuged at 4700 rpm at 4°C for 15 min. The supernatant solution was adjusted to pH 7.0 with 12 M NaOH and filtered sequentially through 53 and 5 μm nylon mesh. Extracts were analyzed for mannitol and total phenolic content.

### Screening of Thermophilic *Clostridia* for Mannitol Utilization

All strains were screened for mannitol utilization by adding mannitol (20 mM) in Hungate tubes (16 mm × 150 mm, ChemGlass, United Kingdom) with an L-G ratio of 1:1. Pressure and optical density were determined after 7 days of incubation where after they were analyzed for end products.

### Fermentation of Mannitol-Extracts From Macro Algae

BM medium was prepared with algal extract diluted to 20 mM mannitol equivalence. Media was prepared in 25 mL serum bottles. Pressure and optical density were determined after 7 days of incubation where after they were analyzed for end products.

### Kinetic Fermentation of Mannitol and Mannitol-Extracts From Macro Algae by *T. pseudoethanolicus*

*Thermoanaerobacter pseudoethanolicus* was cultivated on mannitol (20 mM) and mannitol extracts from macroalgae (equivalent to 20 mM mannitol) in 125 mL serum bottles. Samples were collected over time for 290 h with the most intensive sampling at early stage of the fermentations. Samples were analyzed as previously described.

### Analytical Methods

Hydrogen was analyzed using a Perkin Elmer gas chromatograph equipped with thermo conductivity detector as previously described Orlygsson and Baldursson (2007). Volatile fatty acids and alcohols were analyzed by gas chromatograph (Perkin Elmer Clarus 580) using a FID detector with 30 m DB-FFAP capillary column (Agilent Industries Inc., Palo Alto, CA, United States) as previously described Orlygsson and Baldursson (2007). Pressure was determined using a PendoTech PMAT-DPG Handheld PressureMAT. Mannitol was analyzed using the colorimetric method described by Sanchez (1998). Briefly, 25 μL of diluted sample (less than 5 mM of mannitol) was added to a microplate to which 125 μL of 500 mM formate buffer (pH 3.0) and 75 μL of 5 mM periodic acid. 75 μL of freshly prepared reaction solution (100 mM acetylacetone, 2 M ammonium acetate, 20 mM thiosulfate) was added, the plate briefly mixed at 150 rpm and then incubated at 100°C on a microplate heating block for 2 min. Samples were then read on a Bioscreen C (Growth Curves Ltd., Finland) at 412 nm against a blank prepared as described except substituting reaction solution for distilled water to correct for colored solutions. Standards were prepared from authentic mannitol at a concentration range of 0.5–5 mM.

Total phenolic compounds were analyzed colorimetrically by transferring 250 μL of sample and 1.25 mL of dH_2_O to a microtube to which 125 μL of Folin-Ciocalteu Reagent was added. After 5 min, 375 μL of 20% (w/v) sodium carbonate was added followed by 500 μL of dH_2_O and incubated at ambient temperature for 2 h. Samples were read at 760 nm on a Shimadzu UV-1800 UV-Visible spectrophotometer in a quartz cuvette (*l* = 1 cm) against a water blank. Results are calculated as gallic acid equivalent (GAE) using a standard curve prepared from gallic acid standards (0–100 μg/mL).

Lactate was analyzed spectrophotometrically according to Ingvadottir et al. (2017). Bradford protein was analyzed according to (Bradford, 1976) with modifications. 300 μL of Bradford reagent was added to a microplate well containing 10 μL of sample and shaken for 30 s at 150 rpm on a microplate shaker. Plates were then read at 600 nm on a Bioscreen C (Growth Curves Ltd., Finland) against a water blank. Bovine Serum Albumin was (0.1–1.25 mg/mL) was used as a standard.

Conductivity was determined using a EC 300 portable conductivity meter (YSI Environmental, United States). Viscosity was measured using a Brookfield DV-II+ Pro EXTRA Programmable Rheometer. Proximate analysis of biomass (protein, fat, ash, and carbohydrate content) was conducted according to standard methods.

## Results

Two types of macroalgal biomass were used in present study to investigate its mannitol content and its extraction. The mannitol present in the macroalgal extracts were then tested as a substrate for six genera of thermophilic bacteria.

### Proximate Analysis of Macro Algae

The two macro algae species used in the present investigation *A. nodosum* and *L. digitata* were analyzed for protein, fat, ash, and total carbohydrates. The bulk of the material was greater than 70% (dw) for both algae although the protein and fat content of *A. nodosum* were higher as compared to *L. digitata* but ash and carbohydrate content of the latter were higher (**Table 1**).

**Table 1:** Proximate analysis of *A. nodosum* and *L. digitata* collected from Akureyri and Husavik, Iceland, respectively.

	Brown algae species	
	*A. nodosum*	*L. digitata*
Protein	6.94 ± 0.32	4.03 ± 0.97
Ash	15.60 ± 1.32	18.07 ± 1.07
Fat	5.21 ± 0.69	0.46 ± 0.24
Carbohydrates	72.24 ± 0.92	77.43 ± 0.98

*Values represent the average of triplicate measurements ± SD.*

### Extraction of Mannitol From Macroalgae

#### Ascophyllum nodosum

**FIGURE 1:**
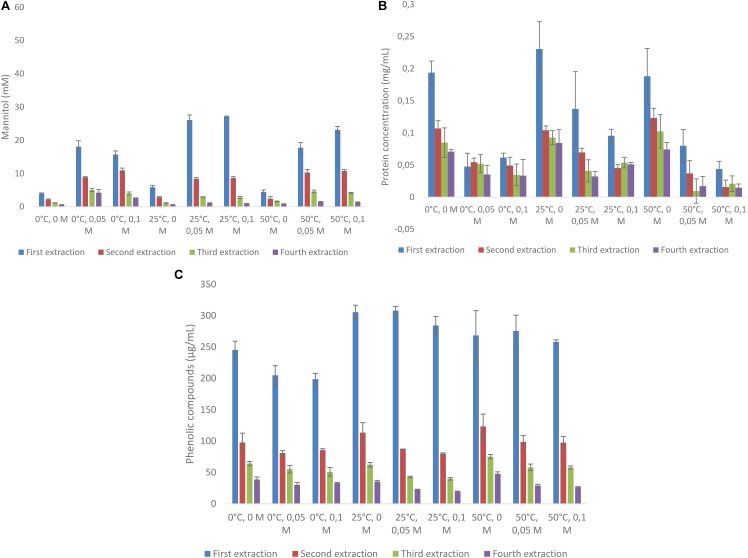
Sequential solid-liquid extraction of mannitol from *Ascophyllum nodosum* at various temperature and HCl concentrations. **(A)** mannitol concentration, **(B)** protein concentration, **(C)** total phenolic compounds as gallic acid equivalent.

Mannitol was extracted from *A. nodosum* with three different concentrations of acid (0, 50, and 100 mM), at three temperatures (0, 25, and 50°C) for four consecutive times. This was also done for protein and total phenolic content (as GAE). The total amounts of mannitol extracted from this macroalgae varied from 7.95 to 39.6 mM (**Figure 1A**). When end concentrations of mannitol extracted are compared with mannitol concentrations obtained after the first extraction, most often more than 50% of mannitol was extracted in this first round (range from 47.2% (0°C, 0.1 M) to 68.5% (25°C, 0.1 M) (**Figure 1A** and **Supplementary Table S2**). Temperature has a clear effect on the amount of mannitol released, with the lowest amounts always found at 0°C and highest at 25°C. Similarly, the effect of acid was profound with little mannitol released without acid but little difference was observed between 50 and 100 mM HCl. Protein extraction from *A. nodosum* is shown in **Figure 1B**. The amount of protein extracted ranged from 0.19 to 0.23 mg/mL with the highest protein amounts observed when the samples were extracted with distilled water at ambient temperature (0.23 mg/mL). Temperature did not greatly impact the amount of protein solubilized during mannitol extraction (**Supplementary Table S3**). The content of phenolic compounds were also analyzed for *A. nodosum* under these same conditions (**Figure 1C**). The only difference with regard to temperature and the concentration of HCl is that lower amounts of phenolic compounds were extracted at 0°C as compared with higher temperatures with and without acid addition (**Supplementary Table S4**).

#### Laminaria digitata

**FIGURE 2:**
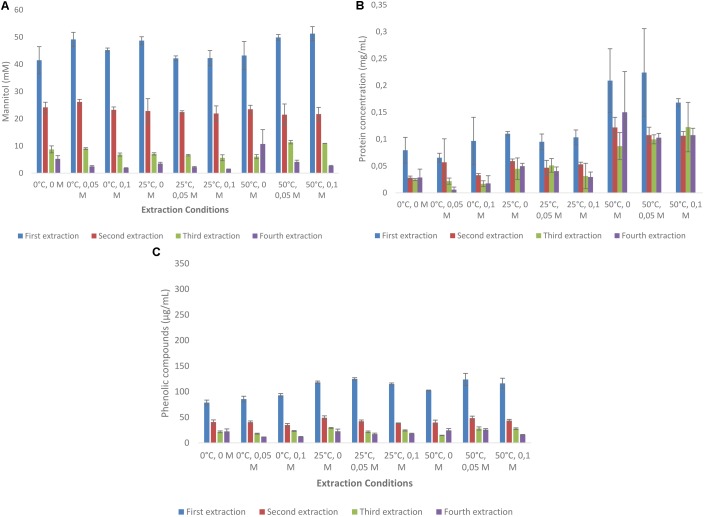
Sequential solid-liquid extraction of mannitol from *Laminaria digitata* at various temperature and HCl concentrations. **(A)** Mannitol concentration, **(B)** protein concentration, **(C)** total phenolic compounds as gallic acid equivalent.

Mannitol extraction from *L. digitata* resulted in much higher concentrations (ranging from 71.3 to 86.9 mM) as compared with *A. nodosum* (**Figure 2A**). First extraction always resulted in more than 50% mannitol extraction when compared with the total amounts extracted (**Supplementary Table S5**). Interestingly, much less variation was found between the use of HCl and temperature as compared with *A. nodosum*. Protein extraction from *A. nodosum* were highest at 50°C with 50 and 100 mM HCl concentrations (0.21–0.22 mg/mL). Much lower concentrations of protein was extracted at other conditions (**Figure 2B**). The concentrations of phenolic compounds from *A. nodosum* (**Figure 2C**) were much lower as compared with *L. digitata* and little difference was between different experimental set up.

### Extraction Kinetics of Mannitol and Phenolics From Macroalgal Meal

**FIGURE 3:**
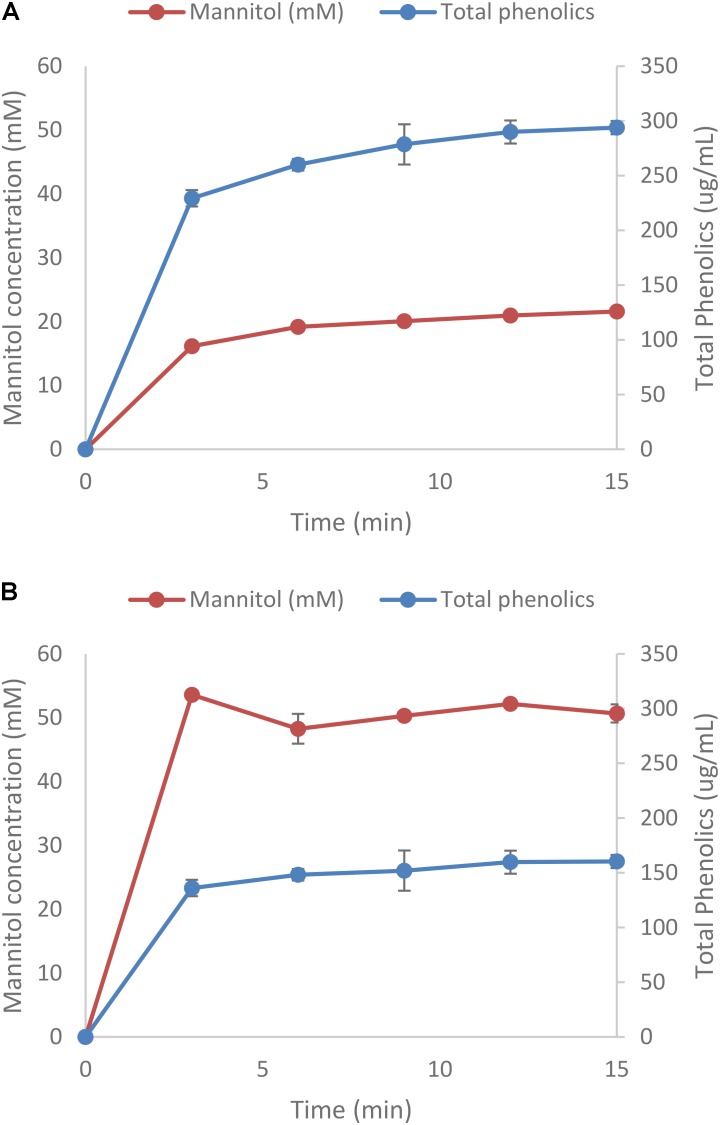
Extraction of mannitol from *Ascophyllum nodosum* **(A)** and *Laminaria digitata* **(B)**. Protein was less then 0.2 mg/mL (data not shown).

In one set of experiments the extraction of both mannitol and phenolic compounds was done kinetically with samples taken every 3 min for 15 min. Conditions chosen were 25°C and 50 mM HCl. **Figures 3A,B** shows that most of the mannitol and phenolic compounds are extracted during the first 3 min of the experiments (more than 75% of the total quantity extracted). As before, mannitol extraction for *L. digitata* were more than twice of that of *A. nodosum* and the concentrations of phenolic compounds were 50% lower (**Supplementary Tables S6, S7**).

### Large Scale Extraction (100 g Scale)

The preparative scale extraction of mannitol from macro algal meal was conducted on a 100 g scale using the optimum conditions (25°C, 50 mM HCl) for each brown algae based on previous experiments as summarized in **Table 2**. As before, the first extraction from both algal meal yielded the highest concentrations of mannitol (19.91 and 65.15 mM, from *A. nodosum* and *L. digitata*, respectively). To reduce the viscosity, CaCl_2_ was added to precipitate any co-extracted alginate.

**Table 2:** Preparative scale extraction of mannitol from brown macro algae.

Macro algae	Extraction no.	Mannitol (mM)	Total phenolics (μg/mL)	Salt (ppt)	Viscosity (cP)
*A. nodosum*	1	19.91 ± 0.25	267.87 ± 0.76	7.4	1950.0
	2	8.02 ± 0.09	114.64 ± 0.5	4.9	24.0
	Total	27.93	382.51		

*L. digitata*	1	65.16 ± 0.52	125.42 ± 2.20	11.2	4580.0
	2	26.12 ± 0.03	65.01 ± 9.18	7.3	1620.0
	Total	91.28	190.43		

*All values represent triplicate analyses of single extraction solutions with SD presented as error bars.*

### Screening of Thermophilic *Clostridia* for Mannitol Utilization

Forty one strains of thermophilic *clostridia* were screened for the degradation and end product formation from 20 mM of mannitol. Most of the strains that were positive on mannitol utilization belong to the genus *Thermoanaerobacter*. Positives within each genera were *Caldanaerobacter* (1 of 5 species), *Caldicellulosiruptor* (2 of 9, both weakly positive), *Thermoanaerobacter* (7 of 16), and *Thermoanaerobacterium* (1 of 7). *T. celere*, and *Caldanaerobius* strains did not utilize mannitol (**Table 3**). End products of mannitol fermentation are presented in **Table 3**.

**Table 3:** End product formation from strains degrading mannitol (20 mM).

		End products (mmol/L)				
Strain	Substrate	Ethanol	Lactate	Acetate	Hydrogen	Carbon balance (%)
*C. subterraneus* subsp. *pacificus* (DSM 12653)	Mannitol	17.3 ± 5.5	2.76 ± 0.81	5.6 ± 1.3	1.7 ± 0.7	64.2
	Control (YE)	0.8 ± 0.2	0.0 ± 0.0	4.8 ± 0.2	5.7 ± 0.7	ND
*T. uzonensis* (DSM 18761)	Mannitol	30.0 ± 1.8	4.33 ± 0.46	3.0 ± 0.1	8.4 ± 1.4	93.5
	Control (YE)	0.8 ± 0.2	0.0 ± 0.0	3.3 ± 0.5	4.0 ± 1.2	ND
*T. sulfurigignens* (DSM 17917)	Mannitol	24.8 ± 1.6	3.89 ± 1.01	4.1 ± 0.8	11.9 ± 1.6	81.8
	Control (YE)	1.7 ± 0.4	0.0 ± 0.0	1.8 ± 0.7	2.2 ± 0.5	ND
*T. siderophilus* (DSM 12299)	Mannitol	33.2 ± 3.2	2.52 ± 1.4	3.0 ± 0.3	9.0 ± 1.7	96.8
	Control (YE)	0.9 ± 0.1	0.0 ± 0.0	2.7 ± 0.7	2.7 ± 0.8	ND
*T. pseudoethanolicus* (DSM 2355)	Mannitol	36.6 ± 6.3	3.40 ± 0.9	3.1 ± 0.2	8.1 ± 1.2	107.7
	Control (YE)	1.4 ± 0.3	0.0 ± 0.0	1.9 ± 0.6	3.2 ± 1.2	ND
*T. italicus* (DSM 9252)	Mannitol	19.7 ± 7.9	6.89 ± 1.48	3.1 ± 0.7	8.5 ± 0.7	74.3
	Control (YE)	0.9 ± 0.3	0.0 ± 0.0	1.4 ± 0.5	2.2 ± 0.6	ND
*T. brockii* subsp *finni* (DSM 3389)	Mannitol	33.4 ± 0.5	3.12 ± 0.24	3.1 ± 0.2	8.9 ± 0.9	99.3
	Control (YE)	1.7 ± 0.5	0.0 ± 0.0	2.7 ± 0.4	3.0 ± 0.8	ND
*T. brockii* subsp *brockii* (DSM 1457)	Mannitol	26.8 ± 6.3	5.27 ± 0.98	3.6 ± 0.3	10.1 ± 1.6	89.2
	Control (YE)	2.2 ± 0.8	0.0 ± 0.0	3.2 ± 0.1	3.6 ± 1.1	ND
*Th. xylanolyticum* (DSM 7097)	Mannitol	18.2 ± 2.2	3.78 ± 0.38	2.6 ± 0.4	3.3 ± 2.8	ND
	Control (YE)	0.6 ± 0.0	0.0 ± 0.0	1.5 ± 0.2	1.4 ± 0.2	ND
*Ca. lactoaceticus* (DSM 9545)	Mannitol	10.0 ± 5.2	5.83 ± 1.23	4.4 ± 0.1	2.2 ± 0.6	38.5
	Control (YE)	0.0 ± 0.0	0.0 ± 0.0	1.0 ± 0.2	1.7 ± 0.2	ND
*Ca. kristjanssonii* (DSM 12137)	Mannitol	8.3 ± 2.3	0.67 ± 0.29	2.4 ± 0.1	3.0 ± 0.2	24.9
	Control (YE)	0.0 ± 0.0	0.0 ± 0.0	2.3 ± 0.2	0.8 ± 0.2	ND

*Values represent the average of triplicate measurements ± SD. C, Caldanaerobacter; T, Thermoanaerobacter; Th, Thermoanaerobacterium; Ca, Caldicellulosiruptor.*

Four of *Thermoanaerobacter* strains are highly ethanologenic producing more than 30 mmol of ethanol from 20 mM of mannitol but the other three produce between 19.7 and 26.8 mM. Other products were lactate, acetate and hydrogen. Only one strain, *C. subterraneus* subsp. *pacificus*, was found positive of the five *Caldanaerobacter* species but produced less ethanol as compared to *Thermoanaerobacter* species. Also, only one of the *Thermoanaerobacterium* strain was found positive, *Th. xylanolyticum* producing 18.2 mM of ethanol, 2.7 mM of acetate, and 3.8 mM of lactate. Two of the *Caldicellulosiruptor* species were weakly positive on mannitol degradation produced low amounts of all end products but above controls.

### Fermentation of Macroalgal Extracts by Selected Strains

**FIGURE 4:**
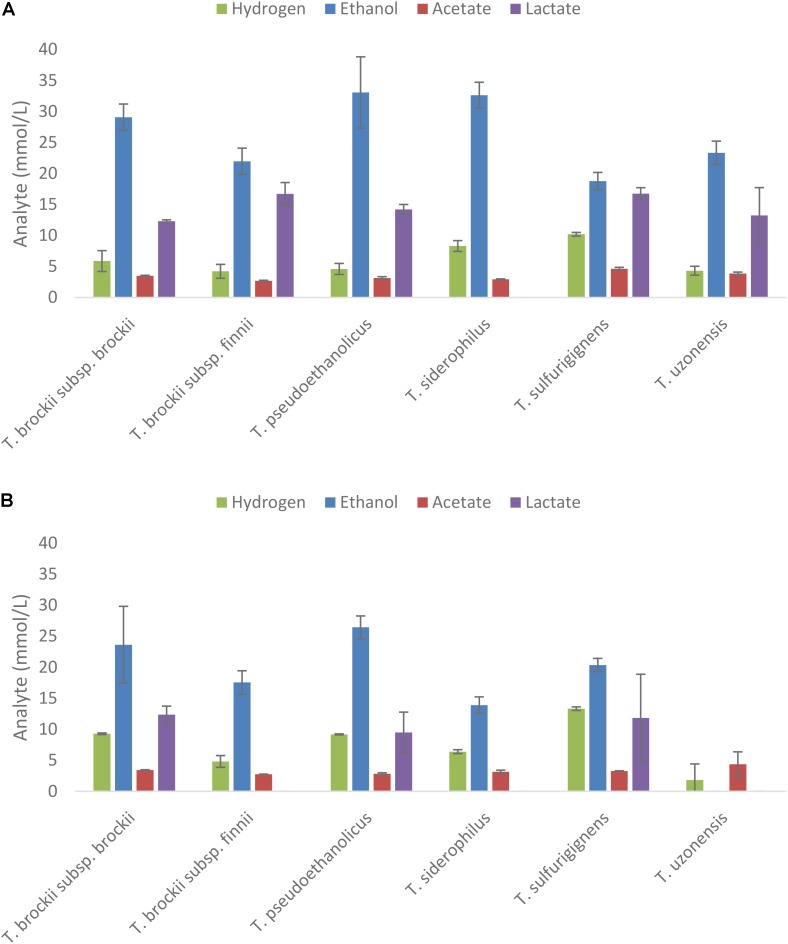
Fermentation products after 5 days from selected thermophilic *Clostridia* on extracts of *Ascophyllum nodosum* **(A)** and *Laminaria digitata* **(B)** adjusted to 20 mM mannitol equivalent. End products were analyzed after 10 days of fermentation; values represent the average of triplicate fermentations with SD presented as error bars.

Strains demonstrating end product formation on mannitol were then grown on extracts of *A. nodosum* and *L. digitata* diluted to a concentration equivalent to 20 mM of mannitol as shown in **Figures 4A,B**, respectively. The only strains showing end-product formation from the extracts belong to the genus of *Thermoanaerobacter*.

Five species (*T. siderophilus, T. pseudoethanolicus, T. brockii* subsp. *finnii, T. brockii* subsp. *brockii, and T. sulfurigignens*) produced considerable amounts of ethanol from both macroalgae species (**Figures 4A,B**). *T. uzonensis* however, only produced ethanol on *A. nodosum* hydrolysate. Ethanol yields (assuming that the only carbon source is mannitol) on the *A. nodosum* extract ranged from 52.5% (*T. sulfurigignens*) to 92.3% (*T. pseudoethanolicus*) and fermentation of the *L. digitata* extract yielded between 52.1% (*T. sulfurigignens*) and 67.6% (*T. pseudoethanolicus*) of the theoretical yield.

### Kinetic Experiment

**FIGURE 5:**
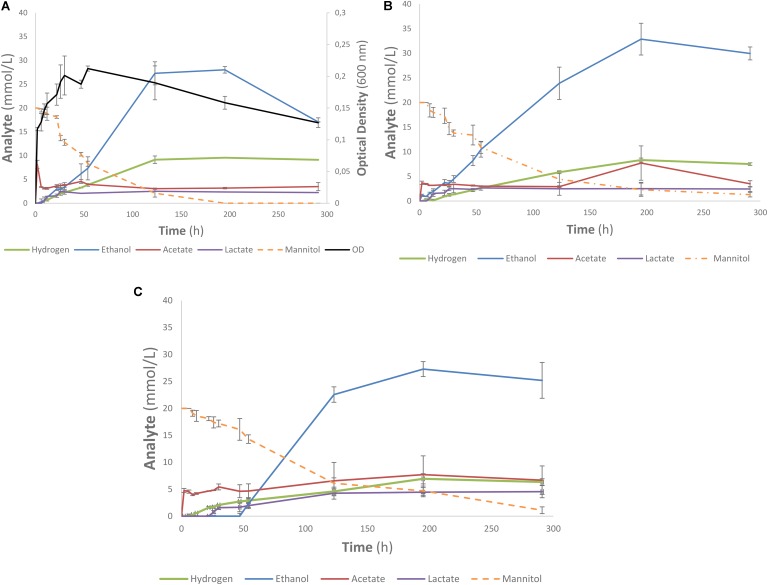
Profile of mannitol-containing media using *Thermoanaerobacter pseudoethanolicus* (DSM 2355). **(A)** 20 mM mannitol, **(B)** *Ascophyllum nodosum* extracted diluted to 20 mM mannitol, **(C)** *Laminaria digitata* extract diluted to 20 mM mannitol. Data points represent the average values of triplicates with SD presented as error bars.

One of the strains yielding high ethanol yields on mannitol and macroalgal extracts was *T. pseudoethanolicus* (**Table 4**). To further investigate mannitol fermentation by this bacterium, kinetic experiments were conducted on glucose and mannitol, as well as the algal extracts from *A. nodosum* and *L. digitata*. **Supplementary Figure S1** shows that glucose is rapidly fermented to ethanol by *T. pseudoethanolicus* reaching 35 mM (87.5% of theoretical) around 48 h. For comparison, fermentation without added carbon source is presented in **Supplementary Figure S2**. The fermentation of mannitol (**Figure 5A**), however, shows the production profile of end-products revealing a rather rapid growth rate and most of the ethanol was already produced within 110 h (final concentration was 28.1 mM which is slightly lower as compared with the screening data on mannitol). Interestingly most of the ethanol is produced after the bacterium reaches stationary growth phase. As previously, the other minor products were acetate, lactate, and hydrogen. During growth on *Laminaria* and *Ascophyllum* extracts similar results were obtained (**Figures 5B,C**) with ethanol being the main end product and acetate and hydrogen were observed below 10 mmol/L.

**Table 4:** Features of kinetic experiments with *Thermoanaerobacter pseudoethanolicus* (DSM 2355) on mannitol and macro algal extracts.

		Ethanol yield		
Substrate	Fermenation time (h)	Yield (%)	Ethanol (g/g substrate)	Volumetric productivity (mM per hour)
Mannitol	123	70.1	0.36	0.29
*A. nodosum* extract	195	82.2	0.42	0.22
*L. digitata* extract	195	68.2	0.35	0.33

## Discussion

### Chemical Composition and Extraction of Mannitol From Macroalgae Biomass

The middle of summer was selected for the sampling period as previous work has shown that the mannitol content peaks around this time in other locations (Adams et al., 2011). While seasonal variation studies on the composition of brown macro algae have not been reported in Iceland, the observed values for protein, fat, and ash were within the ranges previously reported (Indergaard and Minsaas, 1991 and references therein). The bulk of both *A. nodosum* and *L. digitata* collected were carbohydrates (>70% on a dry weight basis) with a large ash fraction (**Table 1**). The collected *L. digitata* contained less than 1% fat which is lower than the range commonly reported but could potentially be attributed to a relatively low quantity of material extracted (approximately 10 g).

Mannitol is an inexpensive carbon source readily extracted from brown macro algae under mild conditions (**Figures 1A, 2A**). Increasing the length of the extraction may result in the co-extraction of phenolics (**Figures 1C, 2C**) which could potentially be inhibitory to fermentative organisms (Daglia, 2012). Relative to the amount of protein present in the algal meal (4–7% w/w), relatively low quantities (<0.2 mg/mL) were extracted under most conditions examined in the present investigation (**Figures 1B, 2B**). The main reason for this difference could be the fact that the proteins present have limited solubility under these conditions or are bound to other components in the matrix. Furthermore, other algal polymers, such as laminarin, fucoidan, alginate, and agar could be co-extracted although this can likely be minimized by using mild conditions and short extraction times as the traditional methodologies for the extraction of these carbohydrates necessitates hot acid extraction (Black et al., 1952; Mateus et al., 1977). For the selective extraction of mannitol, the use of other mineral acids, such as sulfuric acid, maybe more relevant due to HCl’s volatility, especially at elevated temperatures. Kinetic experiments of mannitol extraction under selected conditions reveal that mannitol is rapidly extracted with peak concentrations for the first and second rounds of extraction being achieved in less than 5 min (**Figures 3A,B**). This is more rapid than mannitol extraction reported for other species such as *Macrocystis pyrifera* (Mateus et al., 1977).

In order to reduce the viscosity after large-scale extraction, an extra step involving the addition of calcium chloride to precipitate the alginate was added. This did not adversely affect the concentration of mannitol although increasing the salinity of the solution may adversely affect a number of the organisms being studied.

### Screening of Thermophilic *Clostridia* for Mannitol Utilization

The utilization of mannitol is not routinely reported in characterization papers for most thermophilic *Clostridia* although the type strains of *Caloramator* were not included in this study, *Caloramator boliviensis* and *Caloramator quimbayensis* have been noted to utilize mannitol (Crespo et al., 2012; Rubiano-Labrador et al., 2013).

From the data presented the general trend seems to be that mannitol utilization is largely restricted to the genera of *Thermoanaerobacter* although some strains within *Caldicellulosiruptor* demonstrated weakly positive results evidenced by low ethanol yields (**Table 3**). Generally, the mannitol utilization results reported here are in good agreement with the results which have been previously reported with a few exceptions. The subspecies of *C. subterraneus* have been reported to degrade mannitol qualitatively (Fardeau et al., 2004 and references therein) while in this study only *C. subterraneus* subsp. *pacificus* degrades mannitol with ethanol as the major end product. Furthermore, *T. pentosaceus* was originally reported to utilize mannitol although our results are negative.

Four of the *Thermoanaerobacter* species produce more than 0.4 g ethanol/g mannitol in the present investigation of which the maximum theoretical yields are 0.51 g ethanol/g mannitol (1.5 mol ethanol/mol mannitol). This is comparable with several mesophilic bacteria like *Escherichia coli* and *Zymobacter palmae* which have been reported to produce 0.41 and 0.38 g/g, respectively (Horn et al., 2000; Kim et al., 2011). Other mesophilic microorganism have been reported to produce lower amounts of ethanol, such as *Enterobacter* sp., *Pichia angophorae*, and *Vibrio tritonius* AM2, with 0.29, 0.29, and 0.36 g ethanol/g substrate, respectively. The thermophilic bacterium belonging to Class *Clostridia, Defluviitalea phaphyphila*, has recently been shown to produce high ethanol titers from mannitol with yields of up to 0.44 g/g being reported (Ji et al., 2016a,b).

### Fermentation of Mannitol in Macroalgal Extracts by Selected Strains

Despite positive growth and end product formation on mannitol, strains from the genera of *Caldicellulosiruptor, Caldanaerobacter*, and *Thermoanaerobacterium* did not utilize the mannitol-containing extracts from *A. nodosum* or *L. digitata*. The five mannitol-degrading strains of *Thermoanaerobacter* yielded good ethanol yields from the macro algal extracts, the highest ethanol concentration, 33.1 mM were obtained from *T. pseudoethanolicus* on *A. nodosum* extracts. In general higher ethanol yields were obtained on *Ascophyllum* extracts (between 18.8 and 33.1 mM) compared to *Laminaria* extracts (0.0–26.4 mM). Assuming that mannitol is the only sugar left in the extracts after extraction the ethanol yields are in good correlation with ethanol yields from mannitol only (**Figures 4A,B**). Several studies have been performed using various types of macro algae for ethanol production. When the recently isolated thermophile *Defluviitalea phaphyphila* was cultivated on the alginates from the brown macroalgae *Saccharina japonica* the bacterium produced 2.7 g of ethanol and 3.0 g of acetate from 7.6 g of alginate (Ji et al., 2016a,b). The study by Matsumura et al. (2014) on the brown macroalgae *Saccharina sculpera* using the facultative anaerobe *Vibrio tritonius* resulted in production of 11.5 g of ethanol from 30 g of seaweed hydrolysate. In a study with *E. coli* cultivated on enzyme pretreated hydrolysate of *L. japonica* the maximum ethanol production observed was 0.29 g/g biomass (Kim et al., 2011). Finally, *Saccharomyces cerevisiae* has been reported to produce between 4 and 5 g/L of ethanol in a SSF fermentation of *S. japonica* (Li et al., 2013). It is difficult to compare these results since the concentration of the extracts used, species type and pretreatment methods used vary to a great extent. Clearly however, the best *Thermoanaerobacter* species producing more than 30 mM of ethanol from macroalgal extracts containing 20 mM of mannitol are among the higher yields observed in literature with other microorganism.

As the end products were analyzed after 5 days, it is possible that some strains could exhibit delayed ethanol formation as observed in the kinetic experiments with *T. pseudoethanolicus* on both 20 mM mannitol and the brown algal extracts adjusted to 20 mM of mannitol. Furthermore, the lack of observed end product formation on mannitol-containing algal extracts could be due to inhibition phenomena from co-extracted salts or other inhibitory compounds. The salt concentrations in the macroalgal extracts in our study was 0.5 and 1.1% (**Table 2**). While salt tolerance has not been widely investigated for the type strains of these genera although some data on *Caldicellulosiruptor* species have been reported; *Ca. kristjansson* is inhibited below 0.2% NaCl, *Ca. acetigenus* is inhibited at 0.2% while many other members of the genus only tolerate up to 1% (Onyenwoke et al., 2006; Bing et al., 2015).

### Kinetic Studies of Anntiol and Algal Extract Fermentation

Fermentation of mannitol by *T. pseudoethanolicus* is comparatively slow compared to other substrates (such as glucose, **Supplementary Figure S1**) only reaching a maximum ethanol after 120 h (**Figure 5A**). Ethanol formation continued into the stationary phase which could indicate a bottleneck in feeding mannitol catabolism intermediates into glycolysis pathway. Further work is needed to understand the nature of mannitol utilization by *T. pseudoethanolicus* and to explain why the formation of reduced end products is slower as compared with glucose. It is worth noting that optical density of the culture was approximately 30% higher on glucose as compared with mannitol and three times as high compared with control (yeast extract only) (**Supplementary Figures S1, S2** and **Figure 5A**). The fermentation of the mannitol-containing algal extracts were more slowly fermented to ethanol than with mannitol only (**Figures 5B,C**). The mannitol may have been less accessible to the microorganism due to other compounds in the sample matrix. The optical density was not measured during these fermentations due to the highly turbid nature of the algal extracts. Additionally, fermentation of other compounds present in the extract such as laminarin are possible since some *Clostridia* have been reported to have glucosidases capable of cleaving a β-1,3-*O* glycosidic bond although this capability has not been systematically investigated (Leschine, 2005). As the end product were analyzed after 5 days, it is possible that some strains exhibited delayed ethanol formation as observed in the kinetic experiments with *T. pseudoethanolicus* on both 20 mM mannitol and the brown algae extracts adjusted to 20 mM of mannitol. Furthermore, the lack of observed end product formation on mannitol-containing algae extracts could be due inhibition phenomena from co-extracted salts or other inhibitory compounds.

The slow degradation of mannitol by *T. pseudoethanolicus* could be problematic for commercialization of bioethanol production from macro algae. This could potentially be overcome by increasing the cell density by immobilization, by using continuous culture, as well as the optimization of culture conditions, and genetic engineering of the strain. Furthermore, the utilization of other components of brown macro algae, such as laminarin and alginate, could be approached by hydrolyzing the macro algae using commercially available enzymes. Also, hydrolysates prepared from species known to degrade these polysaccharides could lead to a greater utilization of the biomass perhaps using a cascading biorefinery scheme that couples the removal of higher-value products to the fermentation of lower value fractions such as mannitol.

## Conclusion

Mannitol is a potentially viable source of carbon for the fermentative production of bioethanol by thermophilic anaerobes isolated from diverse environments. Mannitol can be rapidly extracted from *A. nodosum* and *L. digitata* under mild conditions with good yields. The amount of mannitol released from the two species of brown macroalgae varied to a great extent; always lower than 40 mM from *A. nodosum* but higher than 72 mM from *L. digitata*. The amount of protein and phenolic compounds released were also different between macroalgae species (about 300 μg/mL for *A. nodosum* and 100 μg/mL for *L. digitata*). Of 41 thermophilic *Clostridia* species tested for mannitol utilization, 11 were positive mainly species within the genus *Thermoanaerobacter*. Four species showed more than 75% ethanol yields on the sugar alcohol. Six *Thermoanaerobacter* species degraded macroalgal extracts resulting in ethanol as the main end-product. *T. pseudoethanolicus* was the best ethanol producer both from pure mannitol as well as from macroalgae biomass.

## Author Contributions

EI, SS, and TC designed and performed the experiments and analysis. SS and JO drafted the manuscript.

## Conflict of Interest Statement

The authors declare that the research was conducted in the absence of any commercial or financial relationships that could be construed as a potential conflict of interest.
