# Supplementary material for: Fermentation of Mannitol Extracts From Brown Macro Algae by Thermophilic Clostridia
Source: Front Microbiol. 2018 Aug 20;9:1931. doi: 10.3389/fmicb.2018.01931 (PMC6110305; doi:10.3389/fmicb.2018.01931)
Supplement: Supplementary file 1 [file Data_Sheet_1.docx]

**Supplementary Figure 1 –** Degradation of glucose by *Thermoanaerobacter pseudoethanolicus* (DSM 2355). Values represent the average of triplicate fermentations with standard deviation presented as error bars.

**Supplementary Figure 2 –** Fermentation pattern of yeast extract without added external carbon source by *Thermoanaerobacter pseudoethanolicus* (DSM 2355). Values represent the average of triplicate fermentations with standard deviation presented as error bars.

**Supplementary Table 1** – Growth characteristics of type strains of selected thermophilic *Clostridia*; optimum conditions are **bolded** where available.

|  |  |  | **Growth Conditions** | | |  | |
| --- | --- | --- | --- | --- | --- | --- | --- |
| **Strain** | **16S Ascension No.** | **DSM  Number** | T_min_/_opt_/_max_  (°C) | pH_min_/_opt_/_max_ | NaCl (%) |  | **Reference** |
| ***Thermoanaerobacter*** |  |  |  |  |  |  |  |
| *T. acetoethylicus* | L09163 | 2359 | 40/**65**/80 | 5.5/ND/8.5 | ND |  | (Ben-Bassat & Zeikus, 1981; Rainey & Stackebrandt, 1993) |
| *T. brockii* subsp. *brockii* | L09165 | 1457 | 35/**65-70**/85 | 5.5/**6.5-7.5**/9.5 | ND |  | (Zeikus, Hegge, & Anderson, 1979) |
| *T. brockii* subsp. *finnii* | CP002466^a^ | 3389 | 40/**65**/75 | ND/**6.5-6.8**/ND | ND |  | (Cayol et al., 1995; Schmid, et al., 1986) |
| *T. brockii* subsp. *lactiethylicus* | U14330 | 9801 | 40/**55-60**/75 | 5.0/**7.0**/8.5 | 0/**1**/4 |  | (Cayol et al., 1995; Kondratieva, Zacharova, Duda, & Krivenko, 1989) |
| *T. italicus* | AJ250846 | 9252 | 45/**70**/78 | NR/**7.0**/NR | 0/NR/1 |  | (Kozianowski, Canganella, Rainey, Hippe, & Antranikian, 1997) |
| *T. ethanolicus* | L09162 | 2246 | 37/**69**/78 | 4.4/**5.8-8.5**/9.8 | ND |  | (Wiegel & Ljungdahl, 1981) |
| *T. kivui* | L09160 | 2030 | 50/**66**/72 | 5.3/**6.4**/7.3 | ND |  | (Leigh, Mayer, & Wolfe, 1981; Leigh & Wolfe, 1983) |
| *T. mathranii* subsp. *mathranii* | Y11279 | 11426 | 50/**70-75**/75 | 4.7/**7.0**/8.8 | ND^c^ |  | (Larsen, Nielsen, & Ahring, 1997) |
| *T. mathranii* subsp*. Alimentarius* | AY701758 | Not Dep. | 45/ND/70 | NR | NR |  | (Carlier, Bonne, & Bedora-Faure, 2006) |
| *T. pentosaceusus* | GU176611 | 25963 | 50/**70**/80 | 5.5/**7.0**/8.5 | 0/ND/4 |  | (Tomás, Karakashev, & Angelidaki, 2013) |
| *T. pseudoethanolicus* | CP000924^a^ | 2355 | ND/**65**/ND | ND | ND |  | (Onyenwoke, et al., 2007; Zeikus, Ben-Bassat, & Hegge, 1980) |
| *T. siderophilus* | AF120479 | 12299 | 39/**69-71**/78 | 4.8/**6.3-6.5**/8.2 | 0/ND/3.5 |  | (Slobodkin et al., 1999) |
| *T. sulfurigenens* | AF234164 | 17917 | 34/**63-67**/72 | 4.0/**5.0-6.5**/8.0 | ND^b^ |  | (Yong-Jin Lee et al., 2007) |
| *T. sulfurophilus* | Y16940 | 11584 | 55-60 | ND | ND |  | (Bonch-Osmolovskaya et al., 1997) |
| *T. thermocopriae* | L09167 | Not Dep. | 47/**60**/74 | 6.0/**6.5-7.3**/8.0 | ND |  | (Jin, Yamasato, & Toda, 1988) |
| *T. thermohydrosulfuricus* | L09161 | 567 | 37/**67-69**/78 | 5.5/**6.9-7.5**/9.2 | ND |  | (Lee, et al., 1993) |
| *T. uzonensis* | EF530067 | 18761 | 32.5/**61**/69 | 4.2/**7.1**/8.9 | ND |  | (Wagner et al., 2008) |
| *T. wiegelii* | X92513 | 10319 | 38/**65-68**/78 | 5.5/**6.8**/7.2 | ND |  | (Cook, et al., 1996) |
| ***Caldanaerobacter*** |  |  |  |  |  |  |  |
| *C. subterraneus* subsp. *yonseiensis* | AF212925 | 13777 | 50/**75**/85 | 4.5/**6.5**/9.0 | NR |  | (Kim, et al., 2001) |
| *C. subterraneus* subsp. *subterraneus* | AF195797 | 13054 | 40/**65**/75 | 6.0/**7.5**/8.5 | 0/**0**/3 |  | (Fardeau et al., 2004; Fardeau et al., 2000) |
| *C. subterraneus* subsp. *pacificus* | AF174484 | 12653 | 50/**70**/80 | 5.8/**6.8-7.1**/7.6 | ND/**2-2.5**/ND^d^ |  | (Sokolova et al., 2001) |
| *C. subterraneus* subsp. *tengcongensis* | AF209708 | 15242 | 50/**75**/80 | 5,5/**7.0-7.5**/9.0 | 0/**0,2**/2.5 |  | (Xue, et al., 2001) |
| *C. uzonensis* | EF195126 | 18923 | 50/**68-70**/75 | 4.8/**6.8**/8.0 | 0/**0.5**/2 |  | (Kozina, et al., 2010) |
| ***Caldicellulosiruptor*** |  |  |  |  |  |  |  |
| *Ca. changbaiensis* | JX280492 | 26941 | 40/**75**/90 | 58.6/**7.8**/8.6 | 0/NR/1 |  | (Bing et al., 2015) |
| *Ca. saccharolyticus* | L09178 | 8903 | 45/**70**/80 | 5.5/**7.0**/8.0 | NR |  | (Rainey et al., 1994) |
| *Ca. owensis* | U80596 | 13100 | 50/**75**/80 | 5.5/**7.5**/9.0 | 0/NR/1 |  | (Huang, Patel, Mah, & Baresi, 1998) |
| *Ca. bescii* | L09180 | 6725 | 42/**78-80**/90 | 5.0/**7.2**/8.3 | NR |  | (Yang et al., 2010) |
| *Ca. acetigenus* | **AY772476** | 7040 | 50/**65-68**/78 | 5.2/**7.0**/8.6 | 0/NR/0.2 |  | (Nielsen, Mathrani, & Ahring, 1993; Onyenwoke,et al., 2006) |
| *Ca. lactoaceticus* | X82842 | 9545 | 50/**68**/78 | 5.8/**7.0**/8.2 | 0/NR/1 |  | (Mladenovska, Mathrani, & Ahring, 1995) |
| *Ca. kristjanssonii* | AJ004811 | 12137 | 50/**78**/82 | 5.8/**7.0**/8.0 | 0/NR/<0.2 |  | (Bredholt, et al., 1999) |
| *Ca. hydrothermalis* | EF100908 | 18901 | 50/**65**/80 | 6.0/**7.0**/8.0 | 0/NR/1 |  | (Miroshnichenko et al., 2008) |
| *Ca. kronotskiensis* | EF100909 | 18902 | 45/**70**/82 | 6.0/**7.0**/8.0 | 0/NR/1 |  | (Miroshnichenko et al., 2008) |
| ***Thermoanaerobacterium*** |  |  |  |  |  |  |  |
| *Th. thermostercoris* | FM999998 | 22141 | 40/**60**/65 | 5.5/**6.5**/8.0 | 0/**0.5**/2 |  | (Romano et al., 2010) |
| *Th. thermosulfurigenes* | X58351 | 2229 | 60/**55**/75 | 4/**5.5-6.5**/7.6 | ND |  | (Lee et al., 1993) |
| *Th. aotearoense* | X93359 | 10170 | 35/**60-63**/66 | 3.8/**5.2**/6.8 | 0/**0.1**/1 |  | (Liu, et al., 1996; Romano et al., 2010) |
| *Th. thermosaccharolyticum* | M59119 | 571 | 37/**60**/62 | 6.5/**7.8**/8.5 | 0/**0.2**/1 |  | (Collins et al., 1994; McClung, 1935; Romano et al., 2010) |
| *Th. saccharolyticum* | L09169 | 7060 | 45/**60**/68-70 | 5/**6.0**/7.5 |  |  | (Lee et al., 1993) |
| *Th. xylanolyticum* | L09172 | 7097 | 45/**60**/70 | 5.0/**6.0**/7.5 | ND |  | (Lee et al., 1993) |
| *Th. aciditolerans* | AY350594 | 16487 | 37/**55**/68 | 3.2/**5.7**/7.1 | 0/**2.5**/3 |  | (Kublanov et al., 2007; Romano et al., 2010) |
| ***Caldanaerobius*** |  |  |  |  |  |  |  |
| *Caldanaerobius polysaccharolyticus* | U40229 | 13641 | 45/**65-68**/70 | 5.0/**6.8-7.0**/8.0 | ND |  | (Cann, et al., 2001) |
| *Caldanaerobius zeae* | U75993 | 13642 | 37/**65-70**/72 | 3.9/ND/7.9 | ND |  | (Cann et al., 2001) |
| *Caldanaerobius fijiensis* | EF507903 | 17918 | 40/**60-63**/67 | 4.5/**6.8**/8.4 | ND |  | (Lee, et al., 2008) |
| ***Thermobrachium*** *celere* | X99238 | 13655 | 43/**66**/75 | 5.4/**8.2**/9.5 | ND |  | (Engle et al., 1996) |

^a^Whole genome sequence, ^b^Strain tolerates 1 M sodium thiosulfate, ^c^growth not affected at 2% w/v NaCl; ^d^requires seawater for growth; ND-Not determined, NR – Not reported

**References for Supplementary Table 1**

Ben-Bassat, A., & Zeikus, J. G. (1981). Thermobacteroides acetoethylicus gen. nov. and spec. nov., a new chemoorganotrophic, anaerobic, thermophilic bacterium. *Archives of Microbiology*, *128*, 365–370.

Bing, W., Wang, H., Zheng, B., Zhang, F., Zhu, G., Feng, Y., & Zhang, Z. (2015). Caldicellulosiruptor changbaiensis sp . nov ., a cellulolytic and hydrogen-producing bacterium from a hot spring. *International Journal of Systematic and Evolutionary Microbiology*, *65*, 293–297. http://doi.org/10.1099/ijs.0.065441-0

Bonch-Osmolovskaya, E. A., Miroshichenko, L. M., Chernykh, N. A., Kostrikina, N. A., Pikuta, E. V, & Rainey, F. A. (1997). Reduction of elemental sulfute by moderately thermophilic organotrophic bacteria and the description of Thermoanaerobacter sulfurophilus sp. nov. *Mikrobiologiya*, *66*(5), 581–587.

Bredholt, S., Sonne-Hansen, J., Nielsen, P., Mathrani, M., & Ahring, B. K. (1999). Caldicellulosiruptor kristjanssonii sp. nov., a cellulolytic, extremely thermophilic, anaerobic bacterium. *International Journal of Systematic Bacteriology*, (49), 991–996.

Cann, I. K. O., Stroot, P. G., Mackie, K. R., White, B. A., & Mackie, R. I. (2001). Characterization of two novel saccharolytic, anaerobic thermophiles, Thermoanaerobacterium polysaccharolyticum sp. nov. and Thermoanaerobacterium zeae sp. nov., and emendation of the genus Thermoanaerobacterium. *International Journal of Systematic and Evolutionary Microbiology*, *51*(Pt 2), 293–302. Retrieved from http://www.ncbi.nlm.nih.gov/pubmed/11321073

Carlier, J. P., Bonne, I., & Bedora-Faure, M. (2006). Isolation from canned foods of a novel Thermoanaerobacter species phylogenetically related to Thermoanaerobacter mathranii (Larsen 1997): Emendation of the species description and proposal of Thermoanaerobacter mathranii subsp. Alimentarius subsp. Nov. *Anaerobe*, *12*(3), 153–159. http://doi.org/10.1016/j.anaerobe.2006.03.003

Cayol, J. L., Ollivier, B., Patel, B. K., Ravot, G., Magot, M., Ageron, E., … Garcia, J. L. (1995). Description of Thermoanaerobacter brockii subsp. lactiethylicus subsp. nov., isolated from a deep subsurface French oil well, a proposal to reclassify Thermoanaerobacter finnii as Thermoanaerobacter brockii subsp. finnii comb. nov., and an emended descrip. *International Journal of Systematic Bacteriology*, *45*(4), 783–789. http://doi.org/10.1099/00207713-45-4-783

Collins, M. D., Lawson, P. a, Willems, a, Cordoba, J. J., Fernandez-Garayzabal, J., Garcia, P., … Farrow, J. a. (1994). The phylogeny of the genus Clostridium: proposal of five new genera and eleven new species combinations. *International Journal of Systematic Bacteriology*, *44*(4), 812–26. Retrieved from http://www.ncbi.nlm.nih.gov/pubmed/7981107

Cook, G. M., Rainey, F. A., Patel, B. K. C., & Morgan, H. W. (1996). Characterization of a New Obligately Anaerobic Thermophile, Thermoanaerobacter wiegelii sp. nov. *International Journal of Systematic Bacteriology*, *46*(1), 123–127. http://doi.org/10.1099/00207713-46-1-123

Engle, M., Li, Y., Rainey, F., DeBlois, S., Mai, V., Reichert, A., … Wiegel, J. (1996). Thermobrachium celere gen. nov., sp. nov., a rapidly growing thermophilic, alkalitolerant, and proteolytic obligate anaerobe. *International Journal of Systematic Bacteriology*, *46*(4), 1025–33. http://doi.org/10.1099/00207713-46-4-1025

Fardeau, M. L., Magot, M., Patel, B. K. C., Thomas, P., Garcia, J. L., & Ollivier, B. (2000). Thermoanaerobacter subterraneus sp. nov., a novel thermophile isolated from oilfield water. *International Journal of Systematic and Evolutionary Microbiology*, *50*(6), 2141–2149. http://doi.org/10.1099/00207713-50-6-2141

Fardeau, M., Salinas, M. B., L´Haridon, S., Jeanthon, C., Verhé, F., Cayol, J., … Ollivier, B. (2004). Isolation from oil reservoirs of novel thermophilic anaerobes phylogenetically related to Thermoanaerobacter subterraneus : reassignment of T . subterraneus , Thermoanaerobacter yonseiensis , Thermoanaerobacter tengcongensis and Carboxydibrachium pacificu. *International Journal of Systematic and Evolutionary Microbiology*, *54*, 467–474. http://doi.org/10.1099/ijs.0.02711-0

Huang, C., Patel, B. K., Mah, R. A., & Baresi, L. (1998). Caldicellulosiruptor owensensis sp. nov., an anaerobic, extremely thermophilic, xylanolytic bacterium. *International Journal of Systematic and Evolutionary Bacteriology*, *48*, 91–97.

Jin, F., Yamasato, K., & Toda, K. (1988). Clostridium thermocopriae sp. nov., a Cellulolytic Thermophile from Animal Feces, Compost, Soil, and a Hot Spring in Japan. *International Journal of Systematic Bacteriology*, *38*(3), 279–281.

Kim, B. C., Grote, R., Lee, D. W., Antranikian, G., & Pyun, Y. R. (2001). Thermoanaerobacter yonseiensis sp. nov., a novel extremely thermophilic, xylose-utilizing bacterium that grows at up to 85 °C. *International Journal of Systematic and Evolutionary Microbiology*, *51*(4), 1539–1548. http://doi.org/10.1099/00207713-51-4-1539

Kondratieva, E. N., Zacharova, E. V., Duda, V. I., & Krivenko, V. V. (1989). Thermoanaerobium lactoethylicum spec. nov. a new anaerobic bacterium from a hot spring of Kamchatka. *Archives of Microbiology*, *151*, 117–122.

Kozianowski, G., Canganella, F., Rainey, F. a, Hippe, H., & Antranikian, G. (1997). Purification and characterization of thermostable pectate-lyases from a newly isolated thermophilic bacterium, Thermoanaerobacter italicus sp. nov. *Extremophiles*, *1*(4), 171–82. http://doi.org/10.1007/s007920050031

Kozina, I. V., Kublanov, I. V., Kolganova, T. V., Chernyh, N. A., & Bonch-Osmolovskaya, E. A. (2010). Caldanaerobacter uzonensis sp. nov., an anaerobic, thermophilic, heterotrophic bacterium isolated from a hot spring. *International Journal of Systematic and Evolutionary Microbiology*, *60*(6), 1372–1375. http://doi.org/10.1099/ijs.0.012328-0

Kublanov, I. V., Prokofeva, M. I., Kostrikina, N. A., Kolganova, T. V., Tourova, T. P., Wiegel, J., & Bonch-Osmolovskaya, E. A. (2007). Thermoanaerobacterium aciditolerans sp. nov., a moderate thermoacidophile from a Kamchatka hot spring. *International Journal of Systematic and Evolutionary Microbiology*, *57*(2), 260–264. http://doi.org/10.1099/ijs.0.64633-0

Larsen, L., Nielsen, P., & Ahring, B. K. (1997). Thermoanaerobacter mathranii sp. nov., an ethanol-producing, extremely thermophilic anaerobic bacterium from a hot spring in Iceland. *Archives of Microbiology*, *168*(2), 114–119. http://doi.org/10.1007/s002030050476

Lee, Y.-E., Mahendra, J. K., Canyong, L., & Zeikus, Gregory, J. (1993). Taxonomic Distinction of Saccharolytic Thermophilic Anaerobes: Description of Thermoanaerobacterium xylanolyticum gen. nov., sp. nov., and Thermoanaerobacterium saccharolyticum gen. nov., sp. nov.; Reclassification of Thermoanaerobium brockii, Clostridium. *International Journal of Systematic and Evolutionary Microbiology*, *43*(1), 41–51.

Lee, Y.-J., Dashti, M., Prange, A., Rainey, F. a, Rohde, M., Whitman, W. B., & Wiegel, J. (2007). Thermoanaerobacter sulfurigignens sp. nov., an anaerobic thermophilic bacterium that reduces 1 M thiosulfate to elemental sulfur and tolerates 90 mM sulfite. *International Journal of Systematic and Evolutionary Microbiology*, *57*(Pt 7), 1429–34. http://doi.org/10.1099/ijs.0.64748-0

Lee, Y.-J., Mackie, R. I., Cann, I. K. O., & Wiegel, J. (2008). Description of Caldanaerobius fijiensis gen. nov., sp. nov., an inulin-degrading, ethanol-producing thermophilic bacterium from a Fijian hot spring sediment, and reclassification of Thermoanaerobacterium polysaccharolyticum and Thermoanaerobacterium zeae. *International Journal of Systematic and Evolutionary Microbiology*, *58*(3), 666–670. http://doi.org/10.1099/ijs.0.65329-0

Leigh, J. A., Mayer, F., & Wolfe, R. S. (1981). Acetogenium kivui, a new thermophilic hydrogen-oxidizing acetogenic bacterium. *Archives of Microbiology*, *129*(4), 275–280. http://doi.org/10.1007/BF00414697

Leigh, J. A., & Wolfe, R. S. (1983). Acetogenium kivui gen . nov ., sp . nov ., a Thermophilic Acetogenic Bacterium. *International Journal of Systematic Bacteriology*, *33*(4), 866.

Liu, S. Y., Rainey, F. A., Morgan, H. W., Mayer, F., & Wiegel, J. (1996). Thermoanaerobacterium aotearoense sp nov, a slightly acidophilic, anaerobic thermophile isolated from various hot springs in New Zealand, and emendation of the genus Thermoanaerobacterium. *International Journal of Systematic Bacteriology*, *46*(2), 388–396. http://doi.org/10.1099/00207713-46-2-388

McClung, L. S. (1935). Studies on anaerobic bacteria VI. *Journal of Bacteriology*, *29*(2), 189–203.

Miroshnichenko, M. L., Kublanov, I. V, Kostrikina, N. A., Tourova, T. P., Kolganova, T. V, & Bonch-osmolovskaya, E. A. (2008). Caldicellulosiruptor kronotskyensis sp. nov. and Caldicellulosiruptor hydrothermalis sp. nov., two extremely thermophilic, cellulolytic, anaerobic bacteria from Kamchatka thermal springs. *International Journal of Systematic and Evolutionary Microbiology*, *58*, 1492–1496. http://doi.org/10.1099/ijs.0.65236-0

Mladenovska, Z., Mathrani, I. M., & Ahring, B. K. (1995). Isolation and characterization of Caldicellulosiruptor lactoaceticus sp. nov., an extremely thermophilic, cellulolytic, anaerobic bacterium. *Archives of Microbiology*, *163*(3), 223–230.

Nielsen, P., Mathrani, I. M., & Ahring, B. K. (1993). Thermoanaerobium acetigenum spec. nov., a new anaerobic, extremely thermophilic, xylanolytic non-spore-forming bacterium isolated from an Icelandic hot spring. *Archives of Microbiology*, *159*, 460–464.

Onyenwoke, R. U., Kevbrin, V. V., Lysenko, A. M., & Wiegel, J. (2007). Thermoanaerobacter pseudethanolicus sp. nov., a thermophilic heterotrophic anaerobe from Yellowstone National Park. *International Journal of Systematic and Evolutionary Microbiology*, *57*(10), 2191–2193. http://doi.org/10.1099/ijs.0.65051-0

Onyenwoke, R. U., Lee, Y., Dabrowski, S., Ahring, B. K., & Wiegel, J. (2006). Reclassification of Thermoanaerobium acetigenum as Caldicellulosiruptor acetigenus comb . nov . and emendation of the genus description. *International Journal of Systematic and Evolutionary Microbiology*, *56*, 1391–1395. http://doi.org/10.1099/ijs.0.63723-0

Rainey, F. A., Donnison, D. M., Janssen, P. H., Saul, D., Rodrigo, A., Bergquist, P. L., … Morgan, H. W. (1994). Description of Caldicellulosiruptor saccharolyticus gen. nov., sp. nov: An obligately anaerobic, extremely thermophilic, cellulolytic bacterium. *FEMS Microbiology Letters*, *120*, 263–266.

Rainey, F. A., & Stackebrandt, E. (1993). Transfer of the Type Species of the Genus Themobacteroides to the Genus Themoanaerobacter as Themoanaerobacter acetoethylicus ( Ben-Bassat and Zeikus 1981 ) comb . nov ., Description of Coprothemobacter gen . nov ., and Reclassification of Themobacteroide. *International Journal of Systematic Bacteriology*, *43*(4), 857–859.

Romano, I., Dipasquale, L., Orlando, P., Lama, L., d’Ippolito, G., Pascual, J., & Gambacorta, A. (2010). Thermoanaerobacterium thermostercus sp. nov., a new anaerobic thermophilic hydrogen-producing bacterium from buffalo-dung. *Extremophiles*, *14*(2), 233–240. http://doi.org/10.1007/s00792-010-0303-x

Schmid, U., Giesel, H., Schoberth, S. M., & Sahm, H. (1986). Thermoanaerobacter finnii spec. nov., a New Ethanologenic Sporogenous Bacterium. *Systematic and Applied Microbiology*, *8*(1–2), 80–85. http://doi.org/10.1016/S0723-2020(86)80153-9

Slobodkin, A. I., Tourova, T. P., Kuznetsov, B. B., Kostrikina, N. A., Chernyh, N. A., & Bonch-Osmolovskaya, E. A. (1999). Thermoanaerobacter siderophilus sp. nov., a novel dissimilatory Fe(III)-reducing, anaerobic, thermophilic bacterium. *International Journal of Systematic Bacteriology*, *49*(4), 1471–1478. http://doi.org/10.1099/00207713-49-4-1471

Sokolova, T. G., González, J. M., Kostrikina, N. A., Chernyh, N. A., Tourova, T. P., Kato, C., … Robb, F. T. (2001). Carboxydobrachium pacificum gen. nov., sp. nov., a new anaerobic , thermophilic , CO-utilizing marine bacterium from Okinawa Trough. *International Journal of Systematic and Evolutionary Bacteriology*, *51*, 141–149.

Tomás, A. F., Karakashev, D., & Angelidaki, I. (2013). Thermoanaerobacter pentosaceus sp. nov., an anaerobic, extreme thermophilic, high ethanol-yielding bacterium isolated from household waste. *International Journal of Systematic and Evolutionary Microbiology*, *63*, 2396–2404.

Wagner, I. D., Zhao, W., Zhang, C. L., Romanek, C. S., Rohde, M., & Wiegel, J. (2008). Thermoanaerobacter uzonensis sp. nov., an anaerobic thermophilic bacterium isolated from a hot spring within the Uzon Caldera, Kamchatka, Far East Russia. *International Journal of Systematic and Evolutionary Microbiology*, *58*(11), 2565–2573. http://doi.org/10.1099/ijs.0.65343-0

Wiegel, J., & Ljungdahl, L. G. (1981). Thermoanaerobacter ethanolicus gen. nov., spec. nov., a new, extreme thermophilic, anaerobic bacterium. *Archives of Microbiology*, *128*, 343–348.

Xue, Y., Xu, Y., Liu, Y., Ma, Y., & Zhou, P. (2001). Thermoanaerobacter tengcongensis sp. nov., a novel anaerobic, saccharolytic, thermophilic bacterium isolated from a hot spring in Tengcong, China. *International Journal of Systematic and Evolutionary Microbiology*, *51*(4), 1335–1341. http://doi.org/10.1099/00207713-51-4-1335

Yang, S. J., Kataeva, I., Wiegel, J., Yin, Y., Dam, P., Xu, Y., … Adams, M. W. W. (2010). Classification of “Anaerocellum thermophilum” strain DSM 6725 as Caldicellulosiruptor bescii sp. nov. *International Journal of Systematic and Evolutionary Microbiology*, *60*(9), 2011–2015. http://doi.org/10.1099/ijs.0.017731-0

Zeikus, J. G., Ben-Bassat, A., & Hegge, P. W. (1980). Microbiology of methanogenesis in thermal, volcanic environments. *Journal of Bacteriology*, *143*(1), 432–440.

Zeikus, J. G., Hegge, P. W., & Anderson, M. A. (1979). Thermoanaerobium brockii gen. nov. and sp. nov., a new chemoorganotrophic, caldoactive, anaerobic bacterium. *Archives of Microbiology*, *122*(1), 41–48. http://doi.org/10.1007/BF00408044

**Supplemental Table 2 -** Mannitol extraction (mM) from sequential solid-liquid extraction of *Ascophyllum nodosum* powder at various temperatures and hydrochloric acid concentrations. All extractions were performed at a 10% (w/v) solid loading with shaking at 200 rpm for 15 minutes. Data represents the average of triplicates ± standard deviation.

|  |  |  | | | **Extraction condition** | | | | |  | | | | | |
| --- | --- | --- | --- | --- | --- | --- | --- | --- | --- | --- | --- | --- | --- | --- | --- |
|  | **Temp** | **0°C** | | | |  | **25°C** | | | |  | **50°C** | | |  |
| **Number**  **extraction** | **[HCl]** | 0 M | 0.05 M | 0.1 M | |  | 0 M | 0.05 M | 0.1 M | |  | 0 M | 0.05 M | 0.1 M |  |
| 1 |  | 4.0 ± 0.2 | 18.1 ± 1.8 | 15.7 ± 1.1 | |  | 5.9 ± 0.6 | 26.1 ± 1.5 | 27.1 ± 0.2 | |  | 4.4 ± 0.6 | 17.8 ± 1.5 | 23.1 ± 1.0 |  |
| 2 |  | 2.2 ± 0.19 | 8.9 ± 0.2 | 10.9 ± 0.7 | |  | 2.9 ± 0.2 | 8.4 ± 0.4 | 8.7 ± 0.5 | |  | 2.4 ± 0.6 | 10.3 ± 1.0 | 10.7 ± 0.5 |  |
| 3 |  | 1.2 ± 0.1 | 5.1 ± 0.5 | 4.0 ± 0.4 | |  | 1.1 ± 0.1 | 3.0 ± 0.1 | 2.8 ± 0.3 | |  | 1.6 ± 0.1 | 4.6 ± 0.4 | 4.2 ± 0.1 |  |
| 4 |  | 0.6 ± 0.0 | 4.2 ± 1.0 | 2.6 ± 0.1 | |  | 0.6 ± 0.0 | 1.1 ± 0.1 | 1.0 ± 0.0 | |  | 0.8 ± 0.1 | 1.5 ± 0.1 | 1.3 ± 0.1 |  |
| Total Mannitol (mM) |  | 7.95 | 36.21 | 33.30 | |  | 10.43 | 38.56 | **39.58** | |  | 9.22 | 34.18 | 39.47 |  |
| Total Mannitol (g/L) |  | 1.45 | 6.60 | 6.07 | |  | 1.90 | 7.03 | 7.21 | |  | 1.68 | 6.23 | 7.19 |  |
| Total Mannitol (g/g) |  | 0.014 | 0.066 | 0.061 | |  | 0.019 | 0.070 | 0.072 | |  | 0.017 | 0.062 | 0.072 |  |

**Supplemental Table 3** – Protein extraction (µg/mL) from sequential solid-liquid extraction of *Ascophyllum nodosum* powder at various temperatures and hydrochloric acid concentrations. All extractions were performed at a 10% (w/v) solid loading with shaking at 200 rpm for 15 minutes. Data represents the average of triplicates ± standard deviation.

|  |  |  | | | | **Extraction condition** | | |  | | | | | |
| --- | --- | --- | --- | --- | --- | --- | --- | --- | --- | --- | --- | --- | --- | --- |
|  | **Temp** | **0°C** | | |  | **25°C** | | | |  | **50°C** | | |  |
| **Extraction No.** | **[HCl]** | 0 M | 0,05 M | 0,1 M |  | 0 M | 0,05 M | 0,1 M | |  | 0 M | 0,05 M | 0,1 M |  |
|  |  |  |  |  |  |  |  |  | |  |  |  |  |  |
| 1 |  | 193.9 ± 18.1 | 47.6 ± 20.7 | 61.7 ± 6.8 |  | 230.6 ± 42.5 | 137.4 ± 58.0 | 95.6 ± 10.2 | |  | 188.2 ±43.3 | 80.0 ± 25.3 | 43.9 ± 11.9 |  |
| 2 |  | 106.9 ± 12.0 | 54.3 ± 6.4 | 49.3 ± 12.8 |  | 104.0 ± 6.8 | 69.6 ± 6.4 | 45.3 ± 5.3 | |  | 123.3 ±15.1 | 36.7 ± 20.0 | 15.6 ± 10.9 |  |
| 3 |  | 84.8 ± 22.9 | 52.1 ± 13.9 | 34.6 ± 16.9 |  | 92.7 ± 10.9 | 40.8 ± 17.3 | 53.8 ± 8.3 | |  | 102.4 ±26.0 | 9.5 ± 19.00 | 20.9 ± 12.2 |  |
| 4 |  | 70.7 ± 3.8 | 35.1 ± 14.3 | 33.4 ± 25.2 |  | 84.3 ± 20.7 | 32.3 ± 7.5 | 50.9 ± 3.0 | |  | 74.11 ±11.0 | 17.1 ± 15.0 | 14.7 ± 5.7 |  |

**Supplemental Table 4**  – Polyphenol (as gallic acid equivalent, GAE) extraction (µg/mL) from sequential solid-liquid extraction of *Ascophyllum nodosum* powder at various temperatures and hydrochloric acid concentrations. All extractions were performed at a 10% (w/v) solid loading with shaking at 200 rpm for 15 minutes. Data represents the average of triplicates ± standard deviation.

|  |  |  | | | | **Extraction condition** | | | |  | | | | |
| --- | --- | --- | --- | --- | --- | --- | --- | --- | --- | --- | --- | --- | --- | --- |
|  | **Temp** | **0°C** | | |  | **25°C** | | |  | | **50°C** | | |  |
| **Extraction No.** | **[HCl]** | 0 M | 0,05 M | 0,1 M |  | 0 M | 0,05 M | 0,1 M |  | | 0 M | 0,05 M | 0,1 M |  |
|  |  |  |  |  |  |  |  |  |  | |  |  |  |  |
| 1 |  | 245.4 ± 14.0 | 205.1 ± 15.2 | 198.8 ± 8.9 |  | 305.5 ± 11.2 | 307.9 ± 6.5 | 284.5 ± 14.0 |  | | 268.6 ± 39.4 | 275.6 ± 25.2 | 258.4 ± 3.2 |  |
| 2 |  | 97.8 ± 14.9 | 81.1 ± 3.6 | 85.6 ± 2.3 |  | 113.7 ± 15.7 | 87.0 ± 0.4 | 79.8 ± 1.4 |  | | 123.4 ± 19.6 | 98.7 ± 10.0 | 97.7 ± 9.8 |  |
| 3 |  | 64.2 ± 3.4 | 55.1 ± 6.1 | 50.9 ± 6.9 |  | 62.5 ± 3.4 | 42.9 ± 1.2 | 39.8 ± 1.9 |  | | 75.2 ± 3.4 | 58.2 ± 5.2 | 58.0 ± 2.4 |  |
| 4 |  | 38.9 ± 3.8 | 30.3 ± 3.9 | 33.0 ± 1.1 |  | 35.4 ± 1.9 | 22.6 ± 0.7 | 19.4 ± 0.8 |  | | 47.8 ± 3.2 | 28.8 ± 2.3 | 26.5 ± 0.8 |  |

**Supplemental Table 5**  - Mannitol extraction from sequential solid-liquid extraction of *Laminaria digitata* powder at various temperatures and hydrochloric acid concentrations. All extractions were performed at a 10% (w/v) solid loading with shaking at 200 rpm for 15 minutes. Data represents the average of triplicates ± standard deviation.

|  |  |  | | | | **Extraction condition** | | | |  | | | | |
| --- | --- | --- | --- | --- | --- | --- | --- | --- | --- | --- | --- | --- | --- | --- |
|  | **Temp** | **0°C** | | |  | **25°C** | | |  | | **50°C** | | |  |
| **Extraction No.** | **[HCl]** | **0 M** | **0.05 M** | **0.1 M** |  | **0 M** | **0.05 M** | **0.1 M** |  | | **0 M** | **0.05 M** | **0.1 M** |  |
| **1** |  | 41.5 ± 5.0 | 49.2 ± 2.6 | 45.3 ± 0.7 |  | 48.7 ± 1.4 | 42.2 ± 0.9 | 42.9 ± 2.8 |  | | 43.2 ± 5.1 | 49.9 ± 1.0 | 51.3 ± 2.6 |  |
| **2** |  | 24.2 ± 1.9 | 26.2 ± 0.9 | 23.3 ± 1.0 |  | 22.8 ± 4.5 | 22.4 ± 0.5 | 21.9 ± 2.8 |  | | 23.5 ± 1.5 | 21.5 ± 3.9 | 21.7 ± 2.5 |  |
| **3** |  | 8.7 ± 1.3 | 9.0 ± 0.3 | 6.8 ± 0.6 |  | 7.1 ± 0.4 | 6.6 ± 0.3 | 5.7 ± 1.1 |  | | 6.1 ± 0.7 | 11.4 ± 0.6 | 10.9 ± 0.0 |  |
| **4** |  | 5.3 ± 1.2 | 2.5 ± 0.3 | 1.9 ± 0.1 |  | 3.5 ± 0.5 | 2.2 ± 0.2 | 1.5 ± 0.0 |  | | 10.7 ± 5.3 | 4.1 ± 0.6 | 2.6 ± 0.2 |  |

**Supplemental Table 6**  – Protein co-extraction from sequential solid-liquid extraction of *Laminaria digitata* powder at various temperatures and hydrochloric acid concentrations. All extractions were performed at a 10% (w/v) solid loading with shaking at 200 rpm for 15 minutes. Data represents the average of triplicates ± standard deviation.

|  |  |  |  | | | **Extraction condition** | | |  |  |  |  |  |
| --- | --- | --- | --- | --- | --- | --- | --- | --- | --- | --- | --- | --- | --- |
|  | **Temp** | **0°C** | | |  | **25°C** | | | |  | **50°C** | | |
| **Extraction No.** | **[HCl]** | **0 M** | **0.05 M** | **0.1 M** |  | **0 M** | **0.05 M** | **0.1 M** | |  | **0 M** | **0.05 M** | **0.1 M** |
| **1** |  | 79.4 ±24.0 | 65.5 ± 8.3 | 96.6 ± 44.1 |  | 110.0 ± 4.1 | 95.2 ± 14.4 | 103.3 ± 13.7 | |  | 209.0 ± 59.3 | 224.0 ± 81.7 | 168.2 ± 7.2 |
| **2** |  | 28.1 ±3.2 | 56.8 ± 43.8 | 32.9 ± 3.2 |  | 59.2 ± 3.8 | 46.8 ± 13.7 | 53.0 ± 4.1 | |  | 121.9 ± 18.8 | 107.5 ± 14.8 | 106.3 ± 8.0 |
| **3** |  | 24.7 ±1.9 | 21.9 ± 5.7 | 17.1 ± 5.4 |  | 44.9 ± 20.1 | 51.1 ± 12.8 | 31.4 ± 23.6 | |  | 87.1 ± 25.2 | 99.7 ± 8.4 | 122.5 ± 46.1 |
| **4** |  | 28.6 ±15.7 | 6.0 ± 4.8 | 17.5 ± 14.4 |  | 49.7 ± 5.4 | 40.5 ± 7.7 | 29.5 ± 9.3 | |  | 150.1 ± 75.7 | 102.7 ± 8.0 | 107.5 ± 12.8 |

**Supplemental Table 7**  – Total phenolics extraction from sequential solid-liquid extraction of *Laminaria digitata* powder at various temperatures and hydrochloric acid concentrations. All extractions were performed at a 10% (w/v) solid loading with shaking at 200 rpm for 15 minutes. Data represents the average of triplicates ± standard deviation.

|  |  |  | | | **Extraction condition** | | | | |  | | | | |
| --- | --- | --- | --- | --- | --- | --- | --- | --- | --- | --- | --- | --- | --- | --- |
|  | **Temp** | **0°C** | | | |  | **25°C** | | |  | **50°C** | | |  |
| **Extraction No.** | **[HCl]** | **0 M** | **0.05 M** | **0.1 M** | |  | **0 M** | **0.05 M** | **0.1 M** |  | **0 M** | **0.05 M** | **0.1 M** |  |
| **1** |  | 78.7 ± 4.7 | 85.4 ± 5.6 | 92.8 ± 3.4 | |  | 118.3 ± 2.3 | 124.8 ± 2.4 | 114.9 ± 2.0 |  | 102.3 ± 0.7 | 123.7 ± 11.9 | 116.2 ± 10.0 |  |
| **2** |  | 40.6 ± 4.2 | 40.3 ± 2.5 | 34.8 ± 3.0 | |  | 48.9 ± 3.8 | 42.1 ± 2.5 | 38.3 ± 0.6 |  | 39.6 ± 4.8 | 48.2 ± 4.0 | 43.5 ± 2.4 |  |
| **3** |  | 21.6 ± 1.8 | 17.7 ± 0.9 | 23.1 ± 0.9 | |  | 29.0 ± 1.0 | 21.5 ± 1.6 | 24.2 ± 1.1 |  | 14.6 ± 0.4 | 28.1 ± 3.1 | 27.7 ± 1.6 |  |
| **4** |  | 22.4 ± 4.9 | 11.5 ± 0.1 | 11.7 ± 0.6 | |  | 22.8 ± 4.1 | 17.2 ± 1.9 | 17.9 ± 0.9 |  | 24.4 ± 3.5 | 25.8 ± 2.1 | 15.7 ± 0.7 |  |
